# Perceived Experiences of Families of Children with Unilateral Cerebral Palsy in the Implementation of a Task-Specific Intervention in the Home Environment with an Upper Limb Splint: A Qualitative Study

**DOI:** 10.3390/children11101242

**Published:** 2024-10-15

**Authors:** Patricia Roldán-Pérez, Vanesa Abuín-Porras, Isabel Rodríguez-Costa, María Ortiz-Lucas, Pablo Bellosta-López, Almudena Buesa-Estéllez

**Affiliations:** 1 MOTUS Research Group, Universidad San Jorge, Campus Universitario, Autov. A23 km 299, 50830 Zaragoza, Spain; proldan@usj.es (P.R.-P.); pbellosta@usj.es (P.B.-L.); abuesa@usj.es (A.B.-E.); 2 Physiotherapy Department, Faculty of Sport Sciences, Universidad Europea de Madrid, 28670 Madrid, Spain; 3 Area de I+D+i, Fundación DACER, 28702 Madrid, Spain; 4 HIPATIA Research Group, Department of Nursing and Physical Therapy, Faculty of Medicine and Health Sciences, Universidad de Alcalá, 28805 Madrid, Spain; isabel.rodriguezc@uah.es; 5 Education Department, Faculty of Education, Universidad Europea de Madrid , 28670 Madrid, Spain; m.ortiz.lucas@facultyue.es

**Keywords:** unilateral cerebral palsy, qualitative research, parent’s observation, home programs, functional splint

## Abstract

Introduction: Specific home tasks and the use of splints seem to positively affect altered structures and functions, as well as the activities and participation, of children with unilateral cerebral palsy (UCP). However, how did families experience the implementation of these therapies? Objective: To describe the experiences as they were perceived by the families of children with UCP before, during, and after a specific task intervention in the home environment, either with or without upper limb splinting. Methods: A qualitative, descriptive, phenomenological study was conducted in a natural environment. Fourteen families caring for children with UCP who participated in a previous randomized controlled trial were included. Data from unstructured and semi-structured interviews were analyzed through a thematic analysis. Results: Fourteen families (17 parents, age 37–47 years) caring for children with UCP (14 children, ages 6–10 years, 64% female) were interviewed. The following three themes emerged: “The project itself”, in which families explained that they enrolled because of their trust in therapists; “Results obtained”, where the main improvement was the integration of the assisting hand in the body schema; and “Lights and shadows”, where families showed what they learned as positive points and some negative aspects related to the assessments and splints. Conclusions: The perceptions of the parents after the specific task intervention in the home environment showed a greater integration of the most affected side. Nevertheless, although the support of a splint on the hand can have beneficial results in terms of performance, other drawbacks leading to the disuse of the splint were highlighted.

## 1. Introduction

Cerebral Palsy (CP) in childhood greatly impacts the life of the affected child, family dynamics, society, and public policies, as it represents a chronic, complex condition that generates high costs [1]. The child with CP, a non-progressive brain injury, shows posture and movement disorders at both the motor and somatosensory levels, often accompanied by problems with communication, cognition, and/or behavior [1,2]. This situation involves a shift in the family structure, affecting both parents and children. Regarding services for the care of their children, parents identify access to these services, coordination, and the process of care and accompaniment as determining factors for functioning [3]. Covering these factors promotes family empowerment, which aims to provide families with appropriate support that encourages involvement, knowledge, and a sense of control without generating stress or unwanted responsibilities [4]. If the focus is on identifying the needs detected in children after participating in activities of their choice integrated into intensive therapies, the themes that appear are related to (i) learning and showing others the realization of new activities; (ii) experiencing feelings of belonging, solidarity, and friendship; (iii) enjoying doing activities; (iv) participating on their own terms; and (v) transferring to their daily life what they have learned [5].

Regarding adherence to programs developed within the home, a significant relationship has been found between detected family difficulties and non-compliance with home programs [6,7,8,9,10]. Lack of adherence to an intervention may be due to numerous factors, including stress, lack of motivation, disappointment, and fatigue [6]. These circumstances can lead the family to exhaustion and burnout [6]. Parents of children with CP face lengthy interventions, financial problems, home modifications, and the need for special equipment, factors that can contribute to stress [6,8]. Families more easily implement interventions integrated into routines, such as feeding, cleaning, or housework activities. It is proposed that professionals help families contextualize interventions within the home environment and that, in turn, parents develop their own initiatives to implement them [7,10].

Few studies have examined the perception of families regarding adherence and coexistence with upper limb splints. It would appear that a lack of information and training for both the user and the family, as well as feelings of irritability and discomfort, are the main reasons expressed when abandoning the use of a support device [11,12]. Both parents and children with CP express their needs or experiences concerning services and supports, so it is essential to investigate the perceptions of families in the implementation of different interventions, whether these are related to rehabilitation services, programs developed within the home, the development of intensive interventions, or the use of assistive devices [12,13].

Prior to this qualitative study, a randomized clinical trial (RCT, ClinicalTrials.gov ID: NCT03282422) was developed. In this previous RCT, a home program of specific tasks measured in terms of difficulty (activities of daily living and leisure) was implemented with or without the application of a functional upper limb splint for children with unilateral CP (UCP) aged between 5 and 12 years [14]. The conclusions drawn were that although manual function improved in both groups, the use of the functional splint did not appear to improve efficacy over and above the home program. Therefore, the objective of this study is to describe, from a qualitative perspective, the experiences perceived by the families before, during, and after having been a part of the previous study, “Effectiveness of the functional hand splint and specific tasks in the home environment applied to children with cerebral palsy unilateral”.

## 2. Materials and Methods

### 2.1. Design

A phenomenological investigation [15] was conducted within Husserl’s framework to delve into the families’ lived experiences regarding their involvement in the preceding RCT through their firsthand narratives. Within the realm of qualitative inquiry, phenomenology endeavors to grasp the lived experiences of individuals within particular contexts, such as therapeutic interventions, or health behaviors [16]. The objective of phenomenology is to examine phenomena as they manifest in aiming to attain an essential comprehension of human experience. The parents of children with UCP signed the participant information sheet and an informed consent form. The execution of the project was approved by the Ethical Committee for Clinical Research of Aragon (CEICA—Acta Nº 14/2018).

#### Research Team

Three researchers participated in the data collection, transcription, and analysis of the results (PR, a physical therapist and occupational therapist, and MO and AB, physical therapists). AB, who was not involved in the evaluation of the children or their treatment during the experimental phase of the clinical trial, led the development of the interviews. Then, AB and PR preliminarily transcribed and analyzed the data by performing a double encoding process. Finally, the triangulation of the data was carried out by all of the research team members.

### 2.2. Participants

Parents participating in both treatment arms of the previous RCT [14] and who signed the informed consent form to participate in the qualitative study were preselected for the interviews. Exclusion occurred in the case of problems with comprehension or oral communication in the Spanish language. 

#### Sampling Strategies

Two participant selection techniques were applied. First, sampling by purpose was conducted [17,18], which consisted of identifying and selecting a group of persons particularly knowledgeable about or experienced in the phenomenon of interest because they had participated in the previous trial. Second, the theoretical sampling technique was applied to families who could deepen the study’s focus on key aspects that emerged after the preliminary analysis of the first interviews. The sample size was determined by means of the theoretical saturation of the data [19].

### 2.3. Procedure

Four participants were chosen through purpose-based sampling. They underwent unstructured interviews in which the opening question was as follows: “What has been your experience after participating in the previous study…?” 

For the participants chosen through in-depth sampling, semi-structured interviews focused on the key aspects obtained through the preliminary analysis of the unstructured interviews were conducted (Supplementary Materials). The guide of questions was created according to the key aspects obtained through the preliminary analysis of the first four interviews. This guide was piloted among all authors and the participants. The interviews were conducted at the homes of participants or at the rehabilitation clinic the children attend. The mean time for each interview was 60 min.

The interviews were audio-recorded, obtaining 832.54 total minutes of recording.

Lastly, parents answered an online satisfaction questionnaire with two questions based on a Likert scale from 0 to 10 (where 0 is nothing at all and 10 is excellent). The first question asked about their experience as a family in the project, and the second question asked about their experience in the interview.

### 2.4. Data Analysis

Systematic Text Condensation [19] was utilized to analyze the interviews. This method involves an inductive thematic analysis free from any pre-existing theoretical framework. It comprises four main steps: (i) an initial reading to discern preliminary themes for organizing the data; (ii) identifying and categorizing Units of Meaning and delineating the grouping and differentiation of various codes; (iii) engaging in further abstraction using a unit of analysis called Common Meaning Groups, which comprise each grouping, by consolidating aspects representing thematic content; and (iv) synthesizing the condensed content to construct a narrative grounded in the data reflecting both content and meaning.

Coding serves [20] the purpose of streamlining and centralizing specific aspects of the data, thus facilitating the identification of themes and sub-themes. Initially, a comprehensive list of all of the identified or generated units of meaning was compiled during the preliminary analysis. Redundant clusters were then eliminated by assigning numerical codes to each potential theme and sub-theme. Ultimately, all material derived from group discussions and participants’ responses underwent coding.

Qualitative software was not utilized for the analysis. Instead, Office 365 applications, such as Word and Excel, were employed to create transcriptions and the coding grid.

### 2.5. Methodological Rigor

Several methodological procedures to ensure reliability and maintain validity were followed, as described by Lincoln and Guba [21,22,23] (Table 1 and Table 2). More specifically, the research team inspected the transcripts and reviewed them with several informants to ensure credibility and transferability. To maintain dependability, there was continuous communication among team members, and efforts were made to present well-defined and well-described categories. Related to the confirmability, in the case of the informants, we tried to avoid social desirability bias and report the study properly during data collection [24], favoring a relationship of trust and a relaxed and comfortable environment with tact and respect when interviewing the users to obtain the necessary information (e.g., indirect questions, asking for examples, relating it to the context). To ensure catalytic validity, the research team ensured that the study works with and for the community (i.e., “with” by giving voice to participants in this specific context and “for” by introducing strategies for change and improvement of clinical practice). Regarding the triangulation process, it was carried out by the research team during data analysis and also by means of peer checking, as well as triangulation through methods, which was achieved by using the support of quantitative research to discuss the results obtained. From the researchers’ perspective, a bracketing method was carried out by incorporating the process of reflection conducted previously to add this more interpretive part at the end of the descriptive results. In addition, the Standards for Reporting Qualitative Research (SRPQ) [25] and the Consolidated Criteria for Reporting Qualitative Research (COREQ) [26] were followed (Table 1 and Table 2).

## 3. Results

### 3.1. Sociodemographic Data

Data were obtained from 14 families from different areas of Spain. Of these participating families, both parents were present on 3 occasions, and only one of them (mother or father) was present on 11 occasions during the development of the interviews (unstructured and semi-structured). The average age of the 13 mothers was 41 ± 5 years, and the average age of the fathers was 44 ± 3 years. Of the total number of children with UCP (14 children, ages 8 ± 2 years [range of 5–12 years], 64% female, MACS I 42.85%, II 21.42%, III 35.71%; GMFCS I 50%, II 35.71%, III 14.3%), only 5 were present at the interview. The clinical and sociodemographic characteristics of the children and the families were specified by the parents, and they are detailed in Table 3.

### 3.2. Satisfaction Questionnaires

From the data obtained in the satisfaction questionnaires, for the question “Your experience as a family in this project has resulted:”, the mean of the data of the 14 interviews was 9.1 ± 1.0 [range of 7 to 10]. To the question “Rate your experience in this interview:”, the mean of the results was 9.0 ± 1.4 [range of 5 to 10].

### 3.3. Descriptive Results

Regarding the interviews, four were unstructured interviews, while 10 families responded to the semi-structured interview question guide. 

Three themes arose after coding and analyzing the data from the 14 interviews: “The project itself”, “Results obtained”, and “Lights and shadows”.

#### 3.3.1. The Project Itself

This theme consists of three sub-themes: (i) what it consisted of, (ii) why did they decide to participate, and (iii) how they felt about having done it (on the one hand, the parents, and on the other, the children). 

##### What It Consisted Of

The parents described their participation in the project, explaining that it consisted of carrying out an assessment of the child’s bimanual performance at the beginning and later and of integrating tasks to be carried out at home and also leisure-oriented games that involved the use of both hands.

“… they are things that you can carry out in your day to day; like; showering, pouring water into the jug, picking up the dishes, dressing. It is not something that it takes an effort for you to sit down and do it with him… You can organize yourself by saying: I can do this in the morning, this at noon and this at night” (M1, 32 y.o., unstructured interview).

##### Why They Decided to Participate

The main reason participants decided to participate in the previous study was because of the proposal to treat the arm in another way, thus promoting the performance of activities and games that increased family time. In addition, dissemination by the therapists, the fact that it did not present side effects, and the opportunity to increase knowledge about this pathology and its treatment were also other advantages.

“… the study was fine because it wasn’t like ‘come on, stretch her, put her on her knees, let her walk…’ you know? And you also enjoy yourself; even if you are working with her, you enjoy her company” (M6, 32 y.o., semi-structured interview).

##### How the Parents Felt

When faced with completing tasks, parents felt good when their children got them done or frustrated when they could not get through them. The study helped all of them “know the place where they are”, thus encouraging the families to continue working with their children.

“When you see that he achieves something that until now he hasn’t, you celebrate it with great joy” (M11, 36 y.o., semi-structured interview).

“As a mother, I try to be cold minded not to do it myself. It is frustrating to see how she tries and tries again and again and does not succeed.” (M14, 45 y.o., Semi-structured interview)

“… I also like studying because it puts us in our proper place …” (F2, 42 y.o., unstructured interview).

##### How the Children Felt

Parents’ perceptions of how their children feel when doing or not doing tasks and games were collected, and they transferred that feeling of achievement or frustration to a deeper aspect of the experience because children began to be aware that they had to make an effort more than the rest.

“I think he has gained confidence in himself, he showers and brushes his teeth by himself” (M1, 32 y.o., unstructured interview).

“… he begins to ask questions: When will my little hand heal? Why can you do this and I cannot? Why do I have to wear this splint? Or there are activities that he starts not wanting to do because he says he stays “The last one”. … He knows she has to “practice” a little more with his little hand, but he tries hard and always wants to try without hindering” (M11, 36 y.o., semi-structured interview).

#### 3.3.2. Results Obtained

This theme consists of four sub-themes: (i) what can children do now, (ii) maintenance of achievements, (iii) use of “traps”, and (iv) things they cannot do yet. 

##### What Can Children Do Now

Parents explained what tasks their children have managed to integrate into their daily lives.

“He opens the yogurts better, peels the tangerines faster, and the zipper goes up better every time …” (M11, 36 y.o., Semi-structured interview).

“He has integrated several into his daily life such as applying toothpaste, lathering his hands, lathering himself in the shower, opening the cocoa pot, carrying the scooter with both hands” (M14, 45 y.o., Semi-structured interview).

In addition, the interviews reflected the concept of “integration” of the hemiparetic hand from a more global point of view. It can be seen that in most cases, the most important result after the completion of the project was the increased use of the affected hand in the performance of bimanual tasks, whether they wore the splint or not.

“And now the left hand is beginning to form part of everything, of the global …” “… it is functionally slower, but it works.” (M2, 38 y.o., unstructured interview).

“There are some of the things that have remained, she has automated them. And I believe that it is that the things that she sees that she can do for sure, she has totally internalized …” (M4, 41 y.o., unstructured interview).

##### Maintenance of Achievements

This maintenance of achievements seemed to depend on the attitude and personality of the children and the parents when considering the accomplishment of the tasks as a challenge.

“He really likes to improve himself; he doesn’t give up quickly. He likes to try, try, try.” (M1, 32 y.o., unstructured interview).

“Using his right hand is very difficult for him and there are things that frustrate him a lot, and then he doesn’t do it anymore.” (M5, 44 y.o., semi-structured interview).

In the case of parents, their attitude showed how they cope with disability and limitations in the performance of activities by their children. More relaxed attitudes were reflected, which flowed at the same time as the growth of their children. Also, stricter, less permissive ways of acting instead of making an effort to overcome limitations were a constant struggle. Nevertheless, a guilty undertone could be seen in many cases, as they believed they were not doing enough.

“I thought that a mother would be in another way, such as more absorbent … well, if he falls and you have to catch him, then let him fall … because I think they are things he has to live” (M5, 44 y.o., semi-structured interview).

“We are the ones who force her to do certain things. She does nothing by herself, except than sitting on television” (M2, 38 y.o., unstructured interview).

“Maybe because of the rush, I don’t let her develop all that knowledge … And I have a feeling of guilt: ‘‘ oh, if I didn’t do things so much for her, she would also be more independent ‘‘ (M6, 32 y.o., semi-structured interview).

“Our involvement has been less than we would have liked, due to the rhythm of daily life.” (F12, 40 y.o., semi-structured interview).

##### Use of “Traps”

In some cases, parents also described the “traps” or compensatory strategies that children performed to complete the task without involving the use of the affected hand: these resources were easier for them, but they moved away from the objective of the study. Some examples were using the mouth as a grip or performing the bimanual task with only the dominant hand, or parents had to constantly remind children to include their more affected hand in completing tasks.

“The greatest achievement is that to do many of the activities she has stopped using her mouth and now leans on her right hand [affected].” (M14, 45 y.o., semi-structured interview).

“When she has to fix with her left hand [affected], for example, to cut food is not possible, she does it, but with the same hand, that is, she cuts and prick with the right” (M6, 32 y.o., semi-structured interview).

##### Things They Cannot Do Yet

Despite what has been achieved, and even if children use “traps “ and parents insist on the use of the more affected hand to perform tasks, there are still challenges that have not been met. Some examples that still pose a challenge include fastening buttons or zips, combing their hair or putting it in a ponytail, and, for many of them, an activity that is both a form of play and a way of getting around, riding a bicycle.

Sometimes, even though children tried to perform tasks bimanually, they faced the impediment of excess muscle tone that limited mobility and made it difficult to carry out functional activities.

“Some zippers cannot go up properly, tie the laces, open some kind of container, hold the ice cream tub with one hand to eat with the other hand, cut a steak… for example.” (M11, 36 y.o., semi-structured interview).

“We have not yet managed to ride a bike, that is difficult.” (M1, 32 y.o., unstructured interview).

“We try to help him to carry out some tasks, but it blocks both his hand and arm that it is very difficult to unlock them.” “… as soon as you ask him how to use it, it closes, and it is like everything comes together and as a whole.” (M7, 43 y.o., semi-structured interview).

#### 3.3.3. Lights and Shadows

This theme consists of three sub-themes: (i) lights, (ii) halfway, and (iii) shadows. 

##### Lights

The positive aspects that parents highlighted from the study were mainly the following two: the improvements established after participating in the project and the constant help of the therapists in charge of carrying it out. Empathy, closeness, explanations in simple language, and teaching strategies to integrate and maintain these tasks in the routines were what parents value the most. Regarding improvements, the change is highlighted with those achievements that were achieved and maintained.

“Yes, it is true that he stretches his hand more, opens it … the other day, for example, he told me ‘Look mom, I can do that now’” (M5, 44 y.o., semi-structured interview).

“Physiotherapists and occupational therapists talk with us the language we understand, so you go with the document that the rehabilitator gives us, you give it to your physiotherapist or occupational therapist to translate it for you” (M 4, 41 y.o., unstructured interview).

“The way to solve complicated situations in a simple way by following some guidelines and little tricks (given by the investigating therapist).” (F12, 40 y.o., semi-structured interview).

##### Halfway

There were two aspects that the parents assessed both positively and negatively, which were the assessments prior to the task pattern (in which the parents could be present) and the use of the splint (in the case of the children assigned to the experimental group).

“We really liked seeing how she performed the marked tests and how she got involved with them. We have also found out quantitatively how she handled herself with the affected hand.” (M11, 36 y.o., semi-structured interview).

“We have found the evaluations very interesting. We have learned strategies to use on a day-to-day basis so that one can cope more easily in routine situations that may have been more difficult to carry out before” (F12, 40 y.o., semi-structured interview).

Others found negative aspects, such as, for example, filling out a scale in English, as, at the time of the evaluation, it was not yet officially validated in Spanish and updated on the website. Parents had to answer a series of questions in English through a website (www.cheq.se) with the help of a dossier where those same questions were translated into Spanish.

“I had a hard time filling out the test. We do not understand English and the same test in Spanish may take us five minutes” (M7, 43 y.o., semi-structured interview).

The children in the experimental group performed the prescribed tasks carrying a neoprene splint in their most affected hand. There were conflicting opinions about the use of the splint; some children did not mind wearing it, and their parents noticed that they “opened their hands more” due to this fact. However, other children could not bear it. Finally, after completing the study protocol, most children whose families were interviewed did not continue using the splint in their daily lives.

“The problem was that she put it on, but she also took it off by herself; then at school she would say ‘‘ I’m hot ‘‘ and take it off; or ‘‘ I’m going to wash my hands, ‘‘ then she would forget anyway” (M4, 41 y.o., unstructured interview).

“A week after wearing it, she was already happy and has not had any problems. Now she only uses it casually” (M13, 41 y.o., semi-structured interview).

However, there were also positive opinions that can be highlighted.

“The splint on his thumb suited him very well … because afterwards I noticed that he managed to move it … and he also managed to hold more.” (M 4, 41 y.o., unstructured interview).

“… we did notice how his hand was more open.” “Well yes, the finger no longer sticks it.” (F7, 48 y.o., semi-structured interview).

“It has not been a long time since we finished the study, but our intention is that I continue to take her at times of the day.” (M11, 36 y.o., semi-structured interview).

##### Shadows

Parents hardly highlighted negative aspects. Just as some parents had difficulties in the language of the evaluation tests, the assessment sometimes required having to travel from their residence to the city where the study was conducted.

“… it would have made it easier for me if they had come to my house …” (M3, 48 y.o., unstructured interview).

“Difficulties: the trip to Zaragoza for the evaluations.” (M10, 40 y.o., semi-structured interview).

## 4. Discussion

This study examined the experiences perceived by the families before, during, and after participating in the previous study, “Effectiveness of the functional hand splint and specific tasks in the home environment applied to children with unilateral cerebral palsy”, through unstructured and semi-structured interviews. Examination of the feedback provided by the families showed that participation in the project helped parents to “know where they are”, rejoicing at the achievements made by their children but facing the reality that it is difficult to maintain them and confronting all of the challenges that still remain. Overall, the main achievement of the children identified by the families was the “integration” of the hand of the affected half of the body in the performance of bimanual tasks, i.e., children began to use it more in their routines without the need for their parents to have to remind them constantly.

### 4.1. Perception after Implementation of Home Program of Specific Tasks

The perceived benefits of the program of specific tasks integrated into the home reported by the parents bear similarities with those achieved with the “partnership home program” [27,28]. In line with these studies, after implementing the so-called home programs, the parents expressed benefits related to support, realism, flexibility, motivation, generalization of activities, and clarification of roles. Parents also saw these types of interventions as a part of life and in assisting with counseling for the development of activities, transferring to real life, developing routines, quantifying progress, using routines as a means, and increasing family time without perceiving planned tasks as exercises.

Parents are more likely to implement therapeutic programs at home when the programs are included in other daily activities and routines [7]. The fact that parents must find a schedule within the daily routine to carry out the intervention is perceived as a “sacrifice” at a personal, family, work, or leisure level, which is negatively related to the well-being of the family and adherence to the program [7]. This situation coincides with the results obtained.

Novak and Berry indicate that having a program with a small number of tasks that parents feel capable of carrying out safely and therapeutically is essential for the proper development of the intervention [28]. Likewise, in the protocols proposed by Schnackers and Beckers on bimanual training in children with hemiparesis [29], they propose to empower parents by providing them with effective instructions that allow them to organize the demands in a specific way. In the present study, none of the parents expressed a lack of confidence in developing and planning the tasks. These results could be because their reference therapist gave the family the support demanded in planning, changing, and adapting tasks. However, what did emerge was (i) the parents’ frustration when the child did not achieve the task, (ii) the joy when the child reached the goal, and (iii) the struggle not to “help” too much in the development of the tasks.

### 4.2. Family Empowerment

Although the assessments of the current state and performance of the children are sometimes perceived as a “drag” for parents or as an obligation for therapists, they are very necessary to mark the starting point of the intervention [30]. The current study highlights that some key aspects for improving the perception of assessments of parents could be the fact of having good communication and interaction with the therapist. Meanwhile, for therapists, these key aspects could be the provision of resources and the elimination of barriers to carrying them out. Assessment teaches parents to see what their child can do in that context. Furthermore, it has been described that the parents appreciated being able to enter the assessment space and talk with the therapist after the assessment [4].

After analyzing parents as mediators of the intervention, McConnell et al. concluded: “children do well when their families do well, and families do well when they have the resources they need to juggle work and family and care demands” [7]. Similarly to the findings obtained by Monzoor et al. [31], the main achievement identified by the parents in the current study was the integration of the affected hand in bimanual tasks, perceiving that it was more present and that its use was more automated in the development of tasks.

### 4.3. Factors that Affect the Integration of the Affected Hand in Bimanual Tasks

The use of the hand depends not only on abilities but also on the characteristics of the activities and contextual factors [32]. More specifically, a relationship is established between the information the family has integrated about the tasks, the motivation of the children and the parents, and the implementation of the tasks in the routines [32]. In the current study, highly involved and motivated families identified and reinforced the achievements. In contrast, other less constant families showed frustration by not carrying out the activities. These situations may be decisive for the maintenance or not of the achievements over time.

Children use a multitude of strategies for bimanual performance [33]. Among them, children request help from an adult and use compensations, such as bringing objects closer to the body, readjusting the grip on objects with the less affected hand, using wide movements, leaning on surfaces to stabilize themselves, or using their mouths [32]. In this study, some parents indicated that their children had developed strategies to perform different tasks: “they do it their way.” Among the achievements identified after the intervention, some families stated that the support of the affected hand had replaced the use of traps in specific tasks. Thus, it is essential that both the family and the therapists recognize the specificities of each context to maximize the child’s ability to carry out bimanual activities.

The reduction in the feeling bothered parameter in the performance of the task of the Children’s Hand-Use Experience Questionnaire (CHEQ) in both groups of the RCT reflected the clinical relevance of the achievements after the intervention, thus increasing levels of satisfaction in the parents [31]. In addition, parents reported changes related to social behavior (increased confidence and self-assurance), i.e., when the children were able to carry out the proposed tasks, the parents increased their confidence and reduced their frustration to continue doing them [31].

### 4.4. Perception after the Use of Splints

The perceptions of families regarding the use of splints were negative in previous studies using rigid thermoplastic splints [12,33]. In contrast, while the splint used in our study was made of neoprene, the negative aspects of the splint were not related to the difficulty in carrying out activities but rather that the child did not want to use it or that the material made them sweat. Nevertheless, among the positive sensations (lights) manifested by the interviewees, it stands out that the splint did not prevent them from performing any activity and that the hand was even more open. In this sense, the factors related to satisfaction with the use of splints are considered to be (i) the time of use, (ii) the existence of previous training as well as periodic evaluations by professionals, (iii) the child’s adaptation to the orthosis, (iv) the opinion of the family during the prescription, and (v) the feeling of safety while in use [12]. Of the named factors, the most repeated in this work refers to the child’s adaptation to the orthosis, describing both positive and negative adaptations.

### 4.5. Strengths and Limitations

The strengths of this study are centered on the diversity of the profiles of the participants in the interviews, who were families that reside in different parts of Spain with different professions and social statuses. Likewise, the level of affectation of children was diverse (congenital/acquired, MACS, and GMFCS) to enrich the perspectives of parents. Another strength was sharing and contrasting the insights collected with another family participating in the RCT but not in the interviews, which was performed with the intention of establishing the rigor and validity of our qualitative findings [34].

Investigating the children’s opinions would be interesting, and not doing so is a limitation of this study that should be tackled in future projects. For example, this might involve generating participation groups where, in addition to the children, the usual therapists who accompany them in their therapies and/or in the implementation of the programs within the home can also participate. Another limitation was the lack of homogeneity regarding the participation of mothers and fathers in the interviews. The participation rate of the mothers was high, while the fathers participated in only 4 of the 14 interviews, and only one father conducted an interview without the mother’s participation. This imbalance makes it difficult to know the parents’ general perception regarding the project’s development in daily routines.

### 4.6. Future Lines of Research

The previous RCT [14] referred to in this study is included in the so-called home programs, understood by families as an intervention for both parents and children [27]. Therefore, a new analysis will aim to make known the implications of the ICF according to the results obtained in the previous RCT and the perceptions of the families after their participation. Likewise, it is necessary to propose new research investigating the adherence and perception of families when they implement a program based on long-term routines beyond an isolated intervention at one time.

## 5. Conclusions

This study involving 14 families from Spain revealed high satisfaction with the intervention, which focused on improving children’s bimanual performance through daily tasks and leisure activities. Families appreciated the clear communication and support from therapists, which facilitated the integration of rehabilitation tasks into daily life without feeling burdensome. Children showed notable improvements in using their affected hand, although some challenges like fastening buttons and riding bicycles persisted due to muscle tone issues. Mixed reviews were noted regarding the use of splints and the evaluation process, particularly due to language barriers and travel requirements for assessments. Overall, this study underscores the importance of family involvement, continuous evaluation, and adaptable rehabilitation strategies to enhance the empowerment and participation of families in the treatment process.

## Figures and Tables

**Table 1:** Methodological rigor.

Criteria	Description	Technique Used	
Credibility	Confidence in the “truth” of the findings	Research team triangulation	
		Member checking	
Transferability	Showing that the findings are applicable to other contexts	Inspection of transcripts	
		Following a previously established and correct protocol	
		Reviewing the coding of specific parts	
		Communication between team members	
		Presentation of well-defined and well-described topics	
Dependability	Confidence in the “truth” of the findings	Avoiding superficial coding	
		Avoiding the researchers’ interpretations	
		Adding negative cases	
		External auditor	
Confirmability	A degree of neutrality or the extent to which the findings of a study are shaped by the respondents and not researcher bias, motivation, or interest	Research herself/himself	Bracketing
		Participants	Avoiding social desirability bias
			Member checking
		Research team	Triangulation and crystallization
			Peer debriefing
			Catalytic validity

**Table 2:** Bracketing of researchers.

Preliminary theoretical framework	The efficacy of home programs has been demonstrated, as well as the perception of the families after their implementation. However, there is a lack of a purely qualitative approach that reflects how it has been implemented, the time spent, and the involvement in the performance of home tasks with the application or not of functional splinting on the affected hand.
What do you want to investigate?	The experience of families integrating activities that enhance manual function within the child’s routines (ADL and leisure) with or without splinting is being investigated. The aim is to know the usefulness of the resources, the difficulties experienced when modifying routines with new ones, adherence to the intervention at home, and the parents’ opinions after being involved in the process.
Main informants	The main informants will be the parents of children with hemiparesis who have participated in the previous RCT.
Beliefs regarding the experience during the RCT	There were doubts about the adherence of the family in the development of activities during a period of 6 weeks with or without an upper limb splint, as it is not easy to change routines and be consistent in repetitive practice if the family does not see the sense or usefulness of the tasks proposed. A program of low-intensity tasks adapted to the child’s daily life is effective. The development of the program together with the hand splint could boost the improvement of manual function.
Previous experiences	Families often use their holiday time for intensive therapies with a higher or lower cost. Home exercises (stretching, walking on the knees, very specific games, etc.) have been shown not to encourage family adherence, as they require taking extra time they do not have and are perceived as just another chore. Current approaches focus on asking the family about their dynamics, routines, and motivations with the purpose of adapting the routine or modifying it with new strategies that promote the development of skills in the child and the empowerment of the family.
Motivation to develop this research	This study was motivated by the desire to give a voice to the families, to know what they think and the real usefulness of what they have done, and to assess the use of the splint and whether it is feasible to integrate the program into a family’s dynamics. The intention is to be able to analyze the perspective and adherence of the family unit in relation to this type of intervention based on routines integrated in the home and in daily leisure activities.

RCT: randomized clinical trial. ADL: activities of daily living.

**Table 3:** Sociodemographic data.

Participant (Nº)		Age in Years (y.)	Occupation	Participated in the Interview	The Child With UCP Was Present at the Interview	Gender, Age, and Affectation of the Child with Hemiplegia	MACS	GMFCS
1	M	32	Administrative officer	Yes	Yes	Boy, 10 y., congenital, left	II	II
2	M	38	Teacher	Yes	Yes	Girl, 9 y., acquired, left	I	I
	F	42	Operator	Yes				
3	M	48	Physical education teacher	Yes	No	Girl, 10 y., acquired, left	III	II
	F	50	Teacher	No				
4	M	41	Finance manager	Yes	No	Girl, 6 y., acquired, right	III	III
	F	42	Logistics manager	Yes				
5	M	44	Housewife	Yes	No	Boy, 6 y., congenital, right	I	I
	F	42	Welder	No				
6	M	32	Laboratory technician	Yes	No	Girl, 12 y., congenital, left	III	III
	F	33	Truck driver	No				
7	M	43	Housewife	Yes	No	Girl, 6 y., congenital, right	II	II
	F	48	Policeman	Yes				
8	M	47	Architect, state worker	Yes	No	Girl, 6 y., congenital, right	I	II
	F	48	Engineer	No				
9	M	43	Accountant	Yes	No	Boy, 12 y., congenital, right	III	II
	F	43	Engineer	No				
10	M	40	Administrative officer	Yes	No	Boy, 6 y., congenital, right	I	I
	F	43	State worker	No				
11	M	36	Administrative officer	Yes	No	Girl, 5 y., congenital, left	I	I
	F	40	Computer technician	No				
12	M	39	Preschool teacher	No	Yes	Boy, 6 y., congenital, left	III	I
	F	40	Factory technician	Yes				
13	M	41	Pharmacist	Yes	Yes	Girl, 10 y., congenital, right	I	I
	F	44	Technical engineer	No				
14	M	45	Innovation consultant	Yes	Yes	Girl, 9 y., congenital, right	II	I
	F	46	Project manager	No				

M, mother; F, father; y., years; UCP, unilateral cerebral palsy; MACS, Manual Ability Classification System; GMFCS, Gross Motor Function Classification System.

## Data Availability

Data are available upon reasonable request from the authors.

## Supplementary Materials

The following supporting information can be downloaded at https://www.mdpi.com/article/10.3390/children11101242/s1.
