# Supplementary material for: Perceived Experiences of Families of Children with Unilateral Cerebral Palsy in the Implementation of a Task-Specific Intervention in the Home Environment with an Upper Limb Splint: A Qualitative Study
Source: Children (Basel). 2024 Oct 15;11(10):1242. doi: 10.3390/children11101242 (PMC11505963; doi:10.3390/children11101242)

Supplementary Material 1

Mckie Splints- Pediatric Sizing Chart

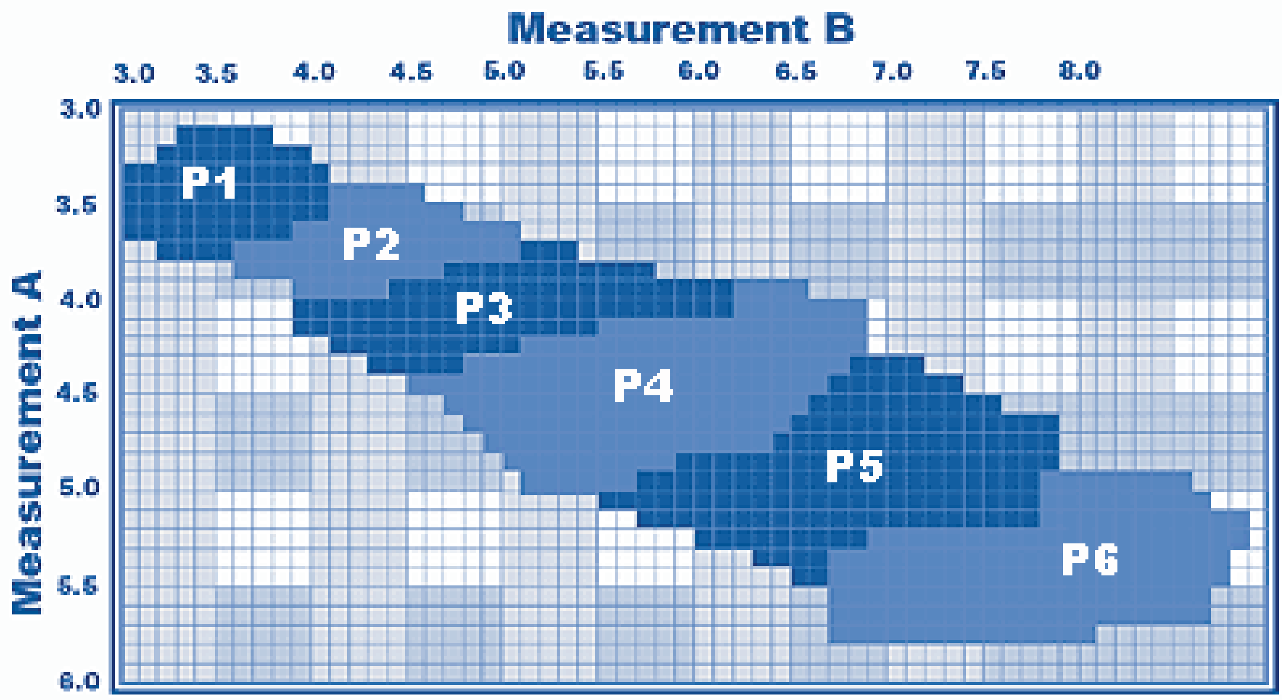

**All measurements are in centimeters*

Only

Mckie A. mckiesplints.com [Internet]. USA: Mckie Splints Pediatric Sizing Chart; 2019 Feb [cited 2019 Jul 7]. Available at: http://cdn.shopify.com/s/files/1/0351/3453/files/pediatric-sizing-chart.pdf?511

Measures

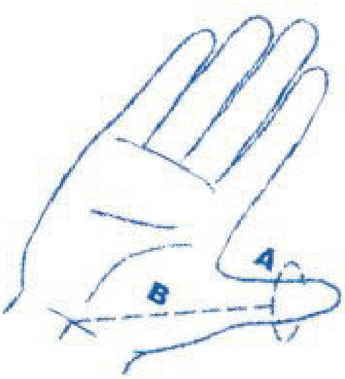

Functional splint

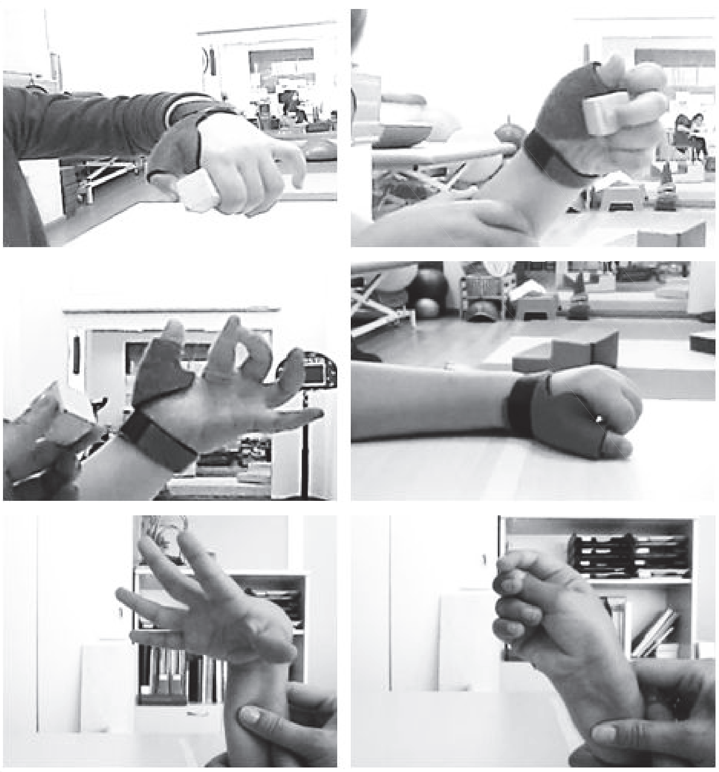

Supplement: Supplementary file 1 [file children-11-01242-s001.zip › children-3245560-supplementary.pdf]
